# Intraoral pressure patterns during swallowing

**DOI:** 10.1007/s00405-012-2299-6

**Published:** 2012-12-13

**Authors:** Petra Santander, Wilfried Engelke, Arno Olthoff, Christiane Völter

**Affiliations:** 1 Dentistry Centre, Department of Maxillofacial Surgery, University of Göttingen, Robert-Koch-Str. 40, 37099 Göttingen, Germany; 2 Phoniatrics and Pedaudiology, Department of Otorhinolaryngology, University of Göttingen, Robert-Koch-Str. 40, 37099 Göttingen, Germany

**Keywords:** Swallowing, Oral phase, Manometry, Negative pressure

## Abstract

Swallowing disturbances are common after neurological disease and oropharyngeal tumor resection. In this case the oral stage is often affected. So far the clinical evaluation of the oral phase is limited. Recently the role of pressure changes during oropharyngeal swallowing has been pointed out, but until now there are not enough data. Thereby 52 healthy adults aged between 20 and 45 years were examined using an oral shield (Silencos^®^, Bredent, Senden, Germany) connected to a digital manometer (GDUSB 1000^®^, Greisinger electronics, Regenstauf, Germany) able to record pressures in a range of 2,000 to −1,000 mbar at a frequency of 1 kHz. Three swallowing conditions were measured: an active bolus intake (ABI) of water, a passive bolus application of a water-bolus (PWA) and a passive application of a gel-bolus (PGA). We found negative pressures with a median value of −278.9 mbar during ABI, of −24.2 mbar during PWA and of −29.4 mbar during PGA. Significant differences in pressure amplitudes and the pressure pattern were observed depending on the kind of bolus application and its consistency. The used test presents a simple and easy to handle method to assess the oral phase of swallowing.

## Introduction

Swallowing, a vital function that secures nutrition and hydration, relies on a complex neuromuscular control and achieves an efficient bolus transport with a protected airway [1]. In healthy subjects this mechanism remains mostly unnoticed during passive swallowing of saliva or during eating and drinking [2, 3]. This process has been described according to Logemann [4] as a sequence of four consecutive stages. Of these, the oral stage is determined primarily by the action of the tongue, whose movement leads to the proper formation of the bolus and the transport through the oral cavity into the pharynx. So far the evaluation of the oral phase of swallowing is limited and still a domain of videofluoroscopy including X-ray exposure and hampered by high subjectivity [5].

With regard to the biofunctional model proposed by Engelke [6] the several participating structures during swallowing can be explained as an interaction of biofunctional compartments and biofunctional valves. According to this model, the interocclusal compartment can be described as the space surrounding the dental arches and is limited anteriorly by the lips. Its posterior limit is given by the linguo-palatal valve, which is formed by the contact between the anterior margin of the tongue and the hard palate. The subpalatinal compartment is located under the palatal vault and its boundaries are the mentioned linguo-palatal valve and the velo-lingual valve, which are defined by the tongue dorsum and soft palate [6]. Thereby swallowing can be understood as a coordinated interaction between the mentioned compartments and the corresponding biofunctional valves following a dynamic pressure gradient. In the past negative pressure amplitudes have already been described in the esophagus and their implication in swallowing has been discussed [7].

Concerning the oral cavity different techniques have been developed so far in order to record intraoral pressure changes such as pressure transducers, balloon air pressure measurements, flush diaphragm pressure transducers, manometers and palatal fitted pressure sensors [8–13]. All these techniques have been applied for the measurement of the contact pressure pattern of the tongue against the palate during the propulsion of the bolus into the pharynx showing a higher pressure gradient in males than in females and in younger versus older persons [14, 15].

So far the intraoral compartment pressure changes during the oral phase of swallowing have not been extensively studied. Thereby the aim of this study was to develop and to apply a non-invasive test on healthy subjects in order to better understand the oral phase of swallowing.

## Materials and methods

The following protocol was approved by the Ethic Committee from the medical school of the University of Goettingen (application number: 24/3/11). All subjects were recruited by volunteer participation responding to a flyer advertisement of the study. All participants gave informed consent to take part in the study.

### Subjects

Intra-oral pressure examination was carried out in 52 subjects (10 males and 42 females) aged from 20 to 45 years. The following selection criteria were applied: no impairment of swallowing or nasal breathing, no abnormal sagittal, vertical and transversal occlusal relationship, no history of head-neck surgery or neurological disease.

### Instruments

**Fig. 1:**
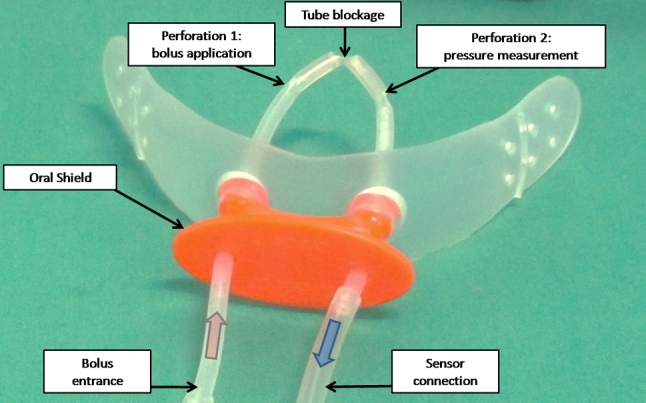
Oral shield composed of a plastic vestibular device and a flexible silicon tube

**Fig. 2:**
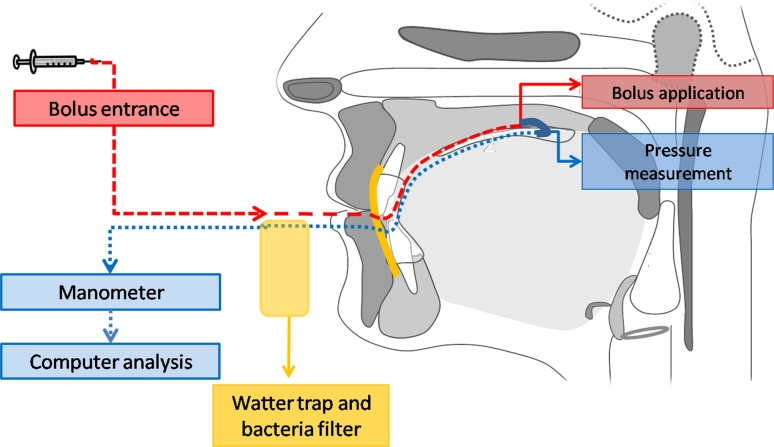
Schematic illustration of the intraoral placement of the used shield

To define the intraoral measurement site and the bolus application the participants were asked to wear a modified oral shield (Fig. 1) (Silencos^®^, Bredent, Senden, Germany). This device is commercially available and used in dental clinical practice for myofunctional treatment. It consists of a silicon shield covering the dental arch buccally, with a plastic lip piece connected to a silicon tube. This tube formed a loop on the dorsal face of the tongue. Two perforations were applied into the tube loop, one to allow water suction from a syringe placed extraorally or for the bolus application via injection. The second perforation was placed for pressure measuring in the subpalatal space. A water trap (Aqua-Knot II Water Trap^®^, General Electric Medical Systems, Wisconsin, USA) and a bacteria filter (Dräger Medical, Lübeck, Germany) were connected in order to ensure precise and safe data recording. The connection from the oral shield to the manometer was given by a pressure pipeline (Gas Sample Line^®^, General Electric Healthcare, Helsinki, Finland). A schematic illustration of the measurement is depicted in Fig. 2.

A piezoresistant relative pressure sensor (GMSD2BR^®^, Greisinger electronics, Regenstauf, Germany) able to record pressures in a range of 2,000 to −1,000 mbar with a resolution of 1 mbar and a frequency of 1 kHz was used. The sensor was connected to a computer-operated manometer (GDUSB 1000^®^, Greisinger electronics, Regenstauf, Germany).

### Examination procedure

All measurements were performed by two investigators in the department of Otorhinolaryngology of the University of Goettingen. The subjects were sitting on a chair, in a comfortable upright position. The experiment was performed under three different study conditions and each condition comprised ten consecutive swallows. Due to the methodic design, normal drinking could not be fully imitated.

### Active bolus intake (ABI)

Subjects were asked to draw water from a syringe over the intraoral silicon loop of the described oral shield. The subjects were told to cumulate enough water to swallow, thereby the collected volume of water differed between individuals. After each attempt the subjects were asked to complete the swallow and to open their mouth.

### Passive water-bolus application (PWA)

A volume of 2 ml of water was applied via the oral shield into the subpalatinal compartment. The subjects were asked to swallow and open their mouth after each attempt.

### Passive gel-bolus application (PGA)

A volume of 2 ml of a gel consistent fluid (Nutilis Aqua^®^, Nutricia Nutilis, Erlangen, Germany) was applied via the oral shield into the subpalatinal compartment. The subjects were asked to swallow and then open their mouth.

During the passive bolus application a volume of 2 ml was chosen with the aim to allowing a subtle swallowing.

### Data analysis

All data were stored in a computer, using the Windows operating software GSOFT-USB^®^ (Greisinger electronics, Regenstauf, Germany). Swallowing was analyzed and registered separately with regard to the pressure amplitude, the curve duration and morphological curve characteristics.

Also a morphological and qualitative description of the curves was performed. Both evaluations, quantitative and a qualitative were made for each single swallow and afterwards correlations were made for the complete trial.

In order to eliminate measurement errors, only pressure changes exceeding ±5 mbar were defined as a swallowing related manometric activity. The amplitude of the curve was measured at the highest point. The duration was measured from a pressure difference of ±5 mbar with regard to the defined measurement error. In this work negative pressure amplitudes were depicted on the upper half of the graphic. First each part of the study was analyzed separately and afterwards correlated between the different test modalities.

## Statistical analysis

The statistical analysis was performed using the MEDAS software (Ch. Grund, Margetshöchheim, Germany). Following tests were performed: Mann–Whitney *U* test, Wilcoxon test, Jones and Boadi-Boateng serial regression and Spearman’s rank correlation. A nominal *p* value of 0.05 and *ρ* > 0.6 were considered statistically significant.

## Results

### Active bolus intake (ABI)

**Fig. 3:**
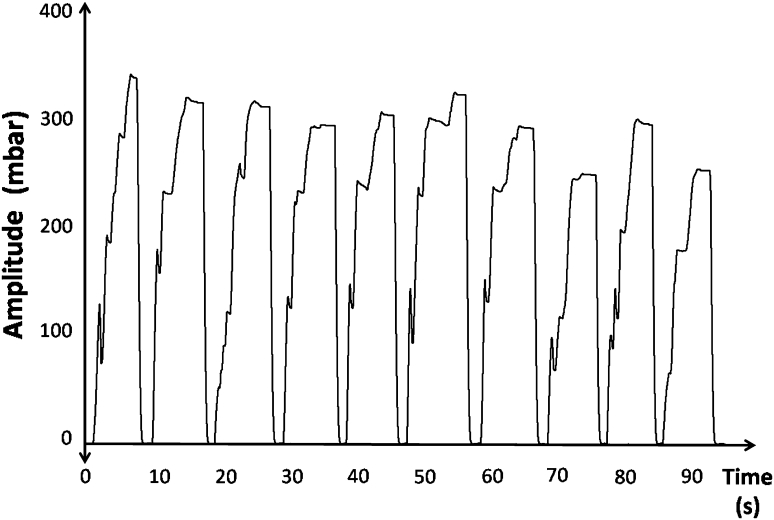
Example of the active bolus intake trial in one patient. Negative pressure amplitudes are depicted on the upper half of the figure. The ten swallow curves show a consistent pattern

After the active intake of water a negative pressure curve was registered in all attempts with a median amplitude of −278.9 mbar (SD: 92.4) and an average duration of 4.9 s (SD: 1.7). The lowest negative pressure amplitude observed was −31 mbar. No positive pressure amplitude was detected. The morphology of the curve showed a high similarity between each single swallow act and also between the different subjects, characterized by a fast rise of the curve, the building of a plateau for a few seconds and a rapid drop of the pressure (Fig. 3).

**Fig. 4:**
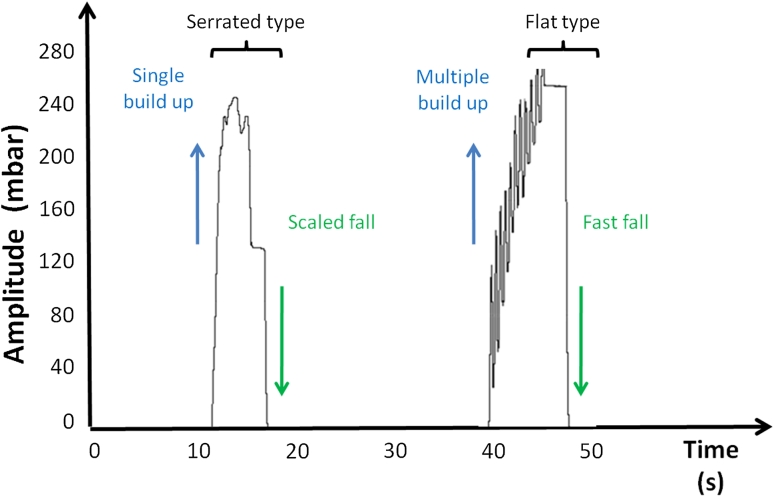
Morphological characteristics of the swallowing curves during the active bolus intake

As depicted in Fig. 4 differences in the curve morphology could be distinguished: 71 % of the cases revealed a fast build up of the negative pressure amplitude defined as a “single build up” with a linear pressure rise. The left 29 % of the subjects had a buildup in repeated steps called “multiple build up”. The top of the curve was characterized in 67.3 % of the subjects by a flat type pressure maintenance and in 25 % of the cases by a serrated type identified by a irregular progression of the curve top. The drop of the pressure was either fast (67.3 %) characterized by a linear pressure drop or scaled (32.7 %) defined as a pressure fall distributed in two steps (Fig. 4). Gender differences were only significant concerning the average duration of the curve, which was shorter in men than in women (*p* = 0.043).

### Passive water-bolus application (PWA)

**Fig. 5:**
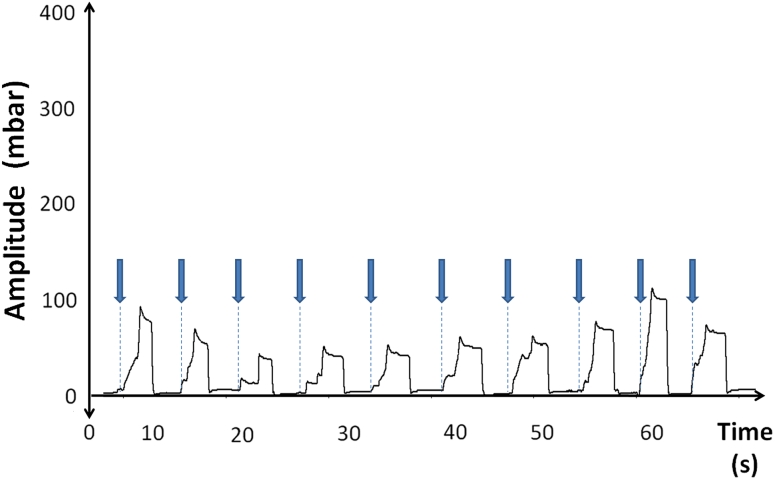
Example of a “wide type curve” during the passive water-bolus application trial in one subject. The *arrows* indicate the application of the bolus

During the application of a bolus of 2 ml of water, also negative pressure was observed during swallowing (Fig. 5). 40 out of the 52 subjects showed a negative pressure gradient in at least eight of ten swallowing attempts. The median amplitude was −24.2 mbar (SD: 23.5), the median curve duration 1.7 s (SD: 1.2). Five out of 52 participants had slight positive pressure amplitudes accompanying the negative pressure peak. The highest positive pressure amplitude observed was 9 mbar.

**Fig. 6:**
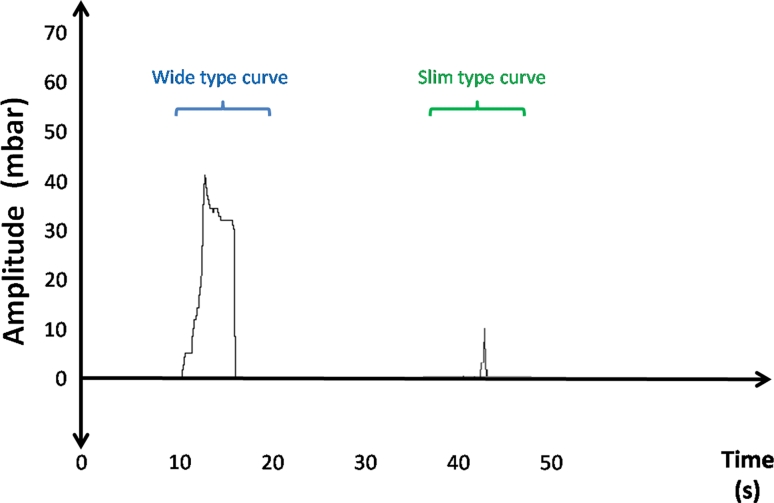
Example of a “wide type curve” and of a “slim type curve” during the passive water-bolus application trial

Regarding the curve morphology a simple curve was observed in all cases, characterized by a single negative pressure rise, followed by a rapid pressure drop. Regarding pressure amplitude and duration of the curve 61.5 % of the subjects showed a curve with high negative pressures and duration of at least 1 s. This type of curve was called “wide type curve” (WTC). The remaining 38.5 % had a narrow or “slim type curve” (STC) with a shorter duration (<1 s) (Fig. 6). Higher negative pressure amplitudes during swallowing were significantly associated with a longer duration of the curve (*p* < 0.001 Jones and Boadi-Boateng serial regression) (*ρ* = 0.7523 and *p* < 0.001 Spearman’s rank correlation). Those subjects with a WTC had a high pressure profile and those with a STC low pressure profile. In this trial, no significant gender differences could be noted.

### Passive gel-bolus application (PGA)

**Fig. 7:**
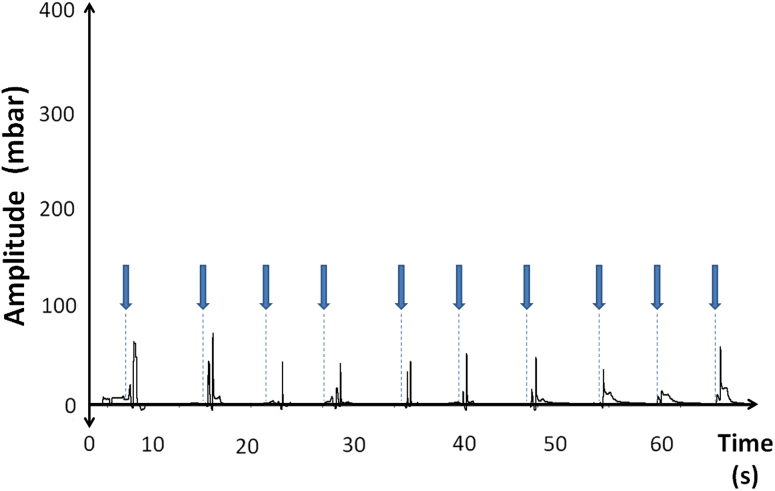
Example of a passive bolus-gel application trial in one subject. The *thick arrows* indicate the application of the bolus. Negative pressure amplitudes are depicted on the upper and positive pressure amplitudes on the lower part of the figure

During the application of a bolus with the consistency of gel, negative pressure amplitudes were observed (Fig. 7). 84.6 % of the subjects showed negative pressure changes in at least eight out of ten swallowing attempts. In this trial, the median pressure amplitude was 29.4 mbar (SD: 29.1) and the median curve duration 1.3 s (SD: 1).

**Fig. 8:**
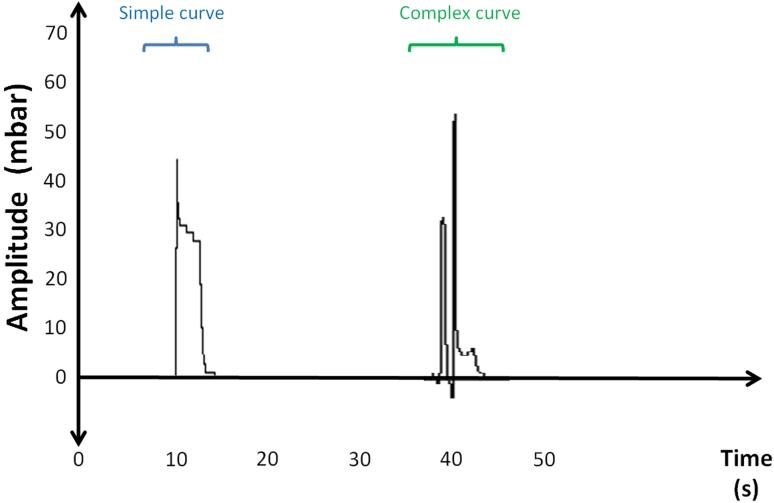
Example of the two different curve types observed during the passive bolus-gel application trial: a “simple curve” with one peak and a “complex curve” with two peaks

During the PGA a unique characteristic was found: the appearance of tight consecutive negative pressure peaks for each single swallows. This so called “complex curve” (CC) characterized by an *M*-typed curve was detected in 67.3 % of the participants. The remaining 32.7 % showed a simple curve (SC) characterized by single peak (Fig. 8).

Concerning the duration and pressure amplitude of the swallowing attempts 30 subjects had a curve duration of at least 1 s, which corresponds to a WTC, 21 subjects had a curve duration shorter than 1 s or a (STC). The complexity of the curve morphology (simple vs. complex) correlated with the amplitude but not with the duration of the peak (Table 1). No significant differences between the genders were observed.

**Table 1:** Median amplitude and duration of the curve in participants with complex and simple curve morphology during the passive gel-bolus application trial

Morphology of the curve	Complex curve (CC)	Simple curve (SC)	*p*
Median amplitude (mbar)	−37.7	−23.6	0.0013*
Median duration (s)	1.5	1.2	0.49

* Indicates a statistically significant difference (*p* after Mann–Whitney *U* test)

Regarding the interindividual variability, the ABI showed a constant pattern in comparison to the passive application of water or gel. This was statistically significant by comparing the standard deviation of the amplitudes (*p* < 0.001, Wilcoxon test).

The median pressure amplitudes of the ABI and of the PGA trial correlated significantly (*ρ* = 0.31, *p* < 0.001 Spearman’s rank correlation). We also found correlations between the morphology of the curve obtained during the PGA and both other trials (*p* after Mann–Whitney *U* test). Subjects with a complex curve during gel application had higher median pressure amplitudes during the ABI trial (−306 vs. −259 mbar, *p* = 0.006) and higher median pressure amplitudes during the PWA trial (−32.5 vs. −21.6 mbar, *p* = 0.062). They also showed a longer duration during the PWA trial (2 vs. 1.3 s, *p* = 0.016).

Regarding the duration of the curve, significant correlations were found between both passive bolus application trials. Subjects with a WTC during the PGA trial had higher median pressure amplitudes (−34.5 vs. −21.3 mbar, *p* = 0.038) and longer curve durations (2 vs. 1.3 s, *p* = 0.021) during the PWA trial, as summarized in Table 2.

**Table 2:** Correlation between the amplitude and the duration of the curve during the passive water-bolus and the passive gel-bolus application

	*ρ*	*p*
Amplitude (mbar)		
Average	0.5133	0.00010*
Median	0.4310	0.00001*
Duration (s)		
Average	0.5622	0.00001*
Median	0.4505	0.00080*

* Indicates a statistically significant difference (*ρ* and *p* after Spearman’s rank correlation)

## Discussion

Negative intraoral pressure amplitudes were present in all three settings. The highest negative amplitude was found during the ABI with a median value of −278.9 mbar, as the participants were asked to collect water by suction from a syringe. The morphology of the curve showed a high inter- and intra-individual stability characterized by a fast and a high rise of negative pressure for a median duration of 5.1 s. This constant and generalized pattern was statistically significant as compared with the pattern recognized during the passive bolus application and might point to the fact that sucking is primal in humans and individual habits might have less influence [16, 17].

Existing information about normal oral suction physiology is poor [18, 19]. Concerning suckling the infant mechanism for milk extraction from the nipple is believed to respond to peristaltic movements of the tongue against the palate and to a vacuum generation [20, 21]. Both mechanisms need a tight interaction between perioral and palatal structures with the related muscles such as lips, cheeks, tongue, soft palate and pharyngeal walls [22]. Structural defects e.g. cleft palate can result in severe feeding problems depending on the severity of the malformation [23, 24].

Studying suction behavior in newborns using ultrasonography and manometric recordings Wein et al. [20] measured negative pressure of about −65 mbar and correlated it with dorsocranial movements of the tongue during velo-lingual contact. Although these pressures differ from those identified by us, morphological similarities can been observed as the curves were characterized by a constant rise of negative pressure, a plateau and a pressure drop.

Concerning adults only one study with a large number of healthy subjects gives information about suction by using a straw to achieve the water intake from a cup [19]. The authors found a median pressure of −195 mbar during a repetitive oral suction swallowing test (ROSS test), lower than the median found in our study (−278.9 mbar). As we did, they also could observe a constant pattern during repetitive forced suction. The observed pressure differences might be due to the age of the collective (18–64 years) and to the fact that the sensor was fixed on the straw [19].

In a following study Nilsson et al. [25] proposed suction as a performance test. They applied the same ROSS test in 100 dysphagic patients 1 week, 1 month and 6 months after stroke. They found higher suction pressures immediately after stroke, suggesting a kind of compensation used by these patients and claimed that this test was able to analyze swallowing disorders.

Interestingly we also measured negative pressure during the passive application of a water and a gel-bolus in the subpalatinal compartment. Compared to the ABI these negative amplitudes were lower and the interindividual differences were larger, possibly underlying the fact that the oral phase of swallowing is highly variable [11, 15, 26].

The amplitude of the measured pressure was similar in both passive bolus application trials (water and gel consistency) (−24.7 mbar during water to −29.4 mbar during gel swallowing), but different concerning the morphology. During the application of a bolus of water two different swallowing patterns were recognized: in 62 % of the studied group a high and long negative pressure rise could be observed, meanwhile in 38 % of the subjects swallowing was associated with slight and brief negative pressure impulses. Whether these differences might be due to swallowing habits such as tongue-thrust due to a persistent infantile swallowing pattern as described by Kittel and Peng et al.—in which the perioral musculature is active during swallowing and the tongue is placed against the central incisors or between the two dental arches—or whether these persons use different swallowing modalities as already pointed out by Dodds et al. [27–29] regarding the tipper and dipper swallowing type, remains so far unknown.

The observed negative pressure during the passive application of a bolus into a closed compartment can only be achieved if the compartment undergoes expansion. Determined by the location of the device used for pressure measuring in this study and regarding the biofunctional model proposed by Engelke [6], the expansion of the subpalatinal compartment takes place, during an effective action of the linguo-palatal and linguo-velar valves. The significance of this negative pressure for the bolus management and the transportation into the pharynx or for triggering the swallowing reflex can so far only be hypothesized. Recently Murata et al. [30] described lower negative pressure during swallowing in the oro- and the hypopharynx in patients suffering of sporadic inclusion body myositis than in normal controls. This low pressure might be the reason for the difficulty in the propulsion of the bolus through the sphincter muscles in these patients.

Due to further development of visual diagnostic procedures, magnetic resonance imaging in real time coupled with manometry might help to better understand the underlying anatomical and physiological differences of the observed swallowing patterns and its implications during deglutition [31–33].

During the passive application of a gel like bolus two notable characteristics could be described: one was the appearance of slight positive amplitudes in 78 % of the participants beside a larger negative pressure gradient associated with swallowing. Secondly, we found the appearance of complex curves, which we only observed during the passive gel application trial and in nearly 70 % of the subjects.

These observed complex curves are similar to those described by Kieser et al. and Kennedy et al. [26, 34] analyzing a small group of test persons (five, respectively six). By use of an individualized rigid palatal plate both authors observed negative pressures in the subpalatinal compartment during the swallowing of 10 ml water. The amplitude of the observed negative pressures was higher than that found in our study. This might be due to relevant methodological differences, the small collective and to the fact that the subjects had to drink from a cup while wearing a palatal plate with attached pressure sensors instead of the passive application into the subpalatinal compartment as in our case. This polyphasic complex pattern characterized by repeated peaks of high negative and slight positive pressure for each swallow could be due to increased tongue movements during swallowing of a bolus with higher consistency [35].

The presented modified oral shield represents a safe method, easily handled, well fitting and well accepted by the patients. As it allows in a simple and objective manner the observation of swallowing capacity, endurance, coordination and rhythmicity, it can serve as a tool for the initial evaluation of swallowing as well as a visual feedback marker in rehabilitation exercises. The presented data collected from a large number of healthy subjects can be used as a reference for further investigations in dysphagic patients.

## Conclusions

Pressure patterns can be measured during swallowing in healthy adults during active and passive bolus intake. Intraoral compartment pressures are predominantly negative and depend on the bolus application and its consistency. The described method represents a simple, safe and clinically applicable test to obtain quantitative and qualitative data of the oral stage of swallowing.
